# Screening for key genes in circadian regulation in advanced atherosclerosis: A bioinformatic analysis

**DOI:** 10.3389/fcvm.2022.990757

**Published:** 2023-01-12

**Authors:** Jiali Yao, Jingyan Liang, Hongliang Li

**Affiliations:** ^1^Institute of Translational Medicine, Medical College, Yangzhou University, Yangzhou, Jiangsu, China; ^2^Jiangsu Key Laboratory of Experimental & Translational Non-Coding RNA Research, Yangzhou University, Yangzhou, Jiangsu, China

**Keywords:** atherosclerosis, circadian rhythm, CCR1, C3aR1, WGCNA

## Abstract

**Background:**

Atherosclerosis (AS) is the most important cardiovascular disease threatening human health, leading to adverse events such as myocardial infarction and stroke. The research on the pathogenesis and causes of AS is being improved step by step, and many factors are associated with AS. However, the relationship between circadian regulation and the pathogenesis of AS is still unclear. Our study identified 2 key genes of circadian regulation in AS by bioinformatics analysis, which provides new perspectives to understand the relationship between circadian rhythm and AS.

**Methods:**

We downloaded samples of early and advanced AS from public databases, screened key genes by weighted gene co-expression network analysis (WGCNA) and Lasso, calculated the immune cell content of the samples using “CIBERSORT,” and analyzed the relationship between key genes and immune cells.

**Results:**

We obtained the most relevant core modules for advanced AS and analyzed the functions of these modules. Two circadian rhythm-related genes were obtained, which influence the immune infiltration of this late AS. ROC curves demonstrated the efficacy of key genes to differentiate between early and advanced AS.

**Conclusion:**

We identified 2 genes most associated with circadian rhythms in advanced AS, whose association with AS has not been elucidated and may become the next therapeutic target.

## 1. Introduction

The global incidence of cardiovascular diseases is increasing year by year and has become a risk factor threatening human health worldwide. WHO predicts that the number of deaths due to cardiovascular diseases will be as high as 23 million in 2030 (1), which is mainly attributed to atherosclerosis (AS). AS is a major risk factor for cardiovascular disease. It is mainly manifested as chronic aseptic inflammation in large vessels, starting with activation of vascular endothelial cells and subsequently undergoing a series of damages such as lipid derivation, fibrosis and calcification, which leads to stenosis and activates an immune inflammatory response (2, 3). AS is associated with various risk factors such as age, dyslipidemia, hypertension, and smoking, leading to myocardial infarction and stroke, as well as disabling peripheral arterial disease (4). Among them, stroke and myocardial infarction are the main causes of death and disability in AS (5). When the lesion causes a stenosis of >50% of the vessel, angina is more likely to be triggered by exercise, exertion, and other conditions. If the lesion’s plaque is unstable or ruptures making the coronary artery completely blocked, it can cause insufficient myocardial blood supply and myocardial infarction (6). Patients with AS at different stages have different degrees of presentation. Early AS is mainly characterized by the accumulation of low-density lipoprotein (LDL) droplets and the production of foam cells, plaques are generally stable and most patients do not have significant symptoms (7). In contrast, in advanced AS, increased vascular inflammation and increased plaque necrosis make plaque rupture or erosion evident, which may be a major cause of acute cardiovascular events (8, 9). Therefore, an in-depth understanding of the mechanisms associated with advanced AS is essential to reduce acute adverse clinical events in patients.

The circadian regulatory system is an important component of the regulation of human metabolic activity, enabling precise regulation of glucose, lipid levels, energy expenditure, and hormone levels in the body over a 24-h period (10, 11). Circadian regulation has also been shown to be involved in the development of several cardiovascular diseases. Previous studies have shown that dysregulation of circadian regulation plays an important role in the progression of hypertension and it’s may become a new direction in the treatment of hypertension (12). Animal experiments have shown that mice with disruption of biological clock-related genes gradually develop disorders of glucose metabolism, including hyperglycemia (13), impaired glucose tolerance (14), and islet β-cell failure (15), suggesting that disruption of circadian rhythms may be involved in the development of type 2 diabetes. A prospective clinical study showed that unhealthy lifestyle and low quality sleep predicted the development of hyperlipidemia and obesity (16) and that shortening sleep duration in children increased the risk of overweight (17). In addition, animal experiments have shown that mutations in genes related to biological clocks are also closely associated with dyslipidemia (18). All these suggest that circadian rhythm disorders in mammals are associated with abnormal lipid metabolism. Also, circadian regulation is involved in a variety of intravascular cellular activities that are essential for maintaining healthy vascular function (19). Recent studies have also demonstrated the important role of circadian regulation in terms of AS, which can be involved in regulating inflammatory and metabolic processes in the vasculature, thus affecting plaque and thrombogenesis (20). In addition, macrophages are involved in all stages of AS and play a key role in the development and progression of advanced AS in particular (21). Apoptosis, passive or accidental necrosis, and secondary necrosis of macrophages in advanced AS fail to perform phagocytosis and accelerate acute cardiovascular events (22, 23). Circadian rhythms can regulate the function of macrophages through related pathways, participating in the development of the disease course of AS (24, 25). As mentioned above, the development of AS is associated with lipid metabolism, inflammatory response, endothelial cell dysfunction, and immune function status. Today there is growing evidence that the regulatory role of the biological clock plays an important role in these processes and that the cytokines involved in AS exhibit circadian oscillations (26). Therefore, exploring the relationship between circadian rhythm-related genes and AS may lead to new ideas for the treatment of AS.

The aim of this study was to identify circadian regulation-related genes in AS. We applied computerized methods to analyze and predict known gene microarray data with standardized and integrated algorithms. Weighted gene co-expression network analysis (WGCNA) is a powerful bioinformatics tool that detects clusters of genes associated with clinical functions, identifies clinically relevant gene markers, and classifies genes with similar clinical functions in the same module. LASSO regression analysis can filter out the core variables among many relevant variables that are most relevant to patient LASSO regression analysis can filter the core variables most relevant to patient prognosis among many relevant variables, optimize the model without reducing the clinical predictive power, and reduce the interference of irrelevant variables on the prediction level. By searching for key genes, the mechanism and biological process of circadian regulation genes in AS will be investigated. The aim is to provide new insights into the intrinsic link between circadian regulation and AS, and to provide a new theoretical basis and research direction for the molecular diagnosis, treatment and prognosis of AS.

## 2. Methods and data

### 2.1. Data download and processing

**FIGURE 1:**
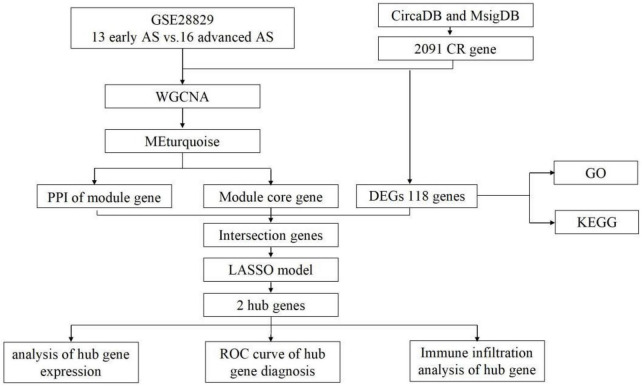
The workflow of this study.

The dataset GSE28829 was downloaded from the GEO database,^1^ which included 13 samples of early AS and 16 samples of advanced AS. 2091 circadian regulation-related genes were downloaded from CircaDB and MsigDB databases. The workflow of this study is shown in Figure 1.

### 2.2. Differential analysis

We extracted circadian regulation-related genes from the dataset GSE28829 and obtained 118 differential genes for early AS and late AS based on | logFC| > 1 and FDR < 0.05, which were presented as heat map and volcano map (R package “pheatmap” and “ggplot2”).

### 2.3. WGCNA to obtain genes associated with advanced AS

Early and late AS-related modules were screened using WGCNA, while core genes associated with late atherosclerosis-related modules were obtained [module membership (MM) > 0.8, Gene significance (GS) > 0.5].

### 2.4. Enrichment analysis

Gene Ontology (GO) and Kyoto Encyclopedia of Genes and Genomes (KEGG) enrichment analyses were performed for the 124 core genes obtained from WGCNA for the most relevant modules of advanced AS, and the R package “ClusterProfiler” was used to plot the relevant histograms and bubble plots.

### 2.5. Screening of key genes

We constructed the PPI network (string)^2^ with 276 genes of the most relevant modules for advanced AS obtained by WGCNA, and screened genes with node number >50 as core genes. To obtain the key circadian regulatory genes for advanced AS, we took the intersection of circadian regulatory genes with early and late differences, the core genes of the most relevant modules with advanced AS, and the core genes screened by the PPI network. Finally, we obtained 11 intersecting genes, and for these intersecting genes, we further used the least absolute shrinkage and selection operator (LASSO) regression algorithm to analyze and obtain 2 key genes most associated with advanced AS. Meanwhile, we used the R package “ggpubr” to draw violin plots to show the differences in expression of key genes in early and advanced AS, and ROC curves to assess their efficacy for the diagnosis of advanced AS.

### 2.6. Immunological correlation

In order to understand the relationship between our candidate key genes and immune cells, we used the software “CIBERSORT” to calculate the content of immune cells in each sample, analyze the correlation between key genes and immune cells, and draw a scatter plot of the correlation, and also use a lollipop plot to summarize the correlation between each immune cell and gene The lollipop plot was also used to summarize the correlation between each immune cell and gene.

## 3. Results

### 3.1. Differential expression of circadian regulation genes

**FIGURE 2:**
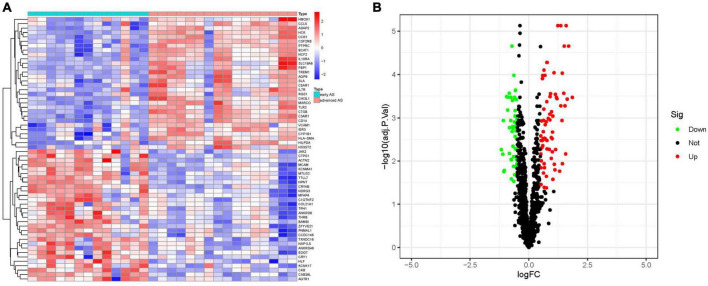
Analysis of differential genes. **(A)** Heat map of differential genes. **(B)** Volcano map of differential genes. (S1 represents early AS, S2 represents advanced AS).

We extracted circadian regulation-related genes from the dataset GSE28829 and compared the differences in circadian regulation-related gene expression between 13 patients with early AS and 16 patients with advanced AS, and obtained 118 differentially expressed genes (Figure 2A), and most of these differentially expressed genes were highly expressed in advanced AS (Figure 2B).

### 3.2. WGCNA to obtain genes associated with advanced AS

**FIGURE 3:**
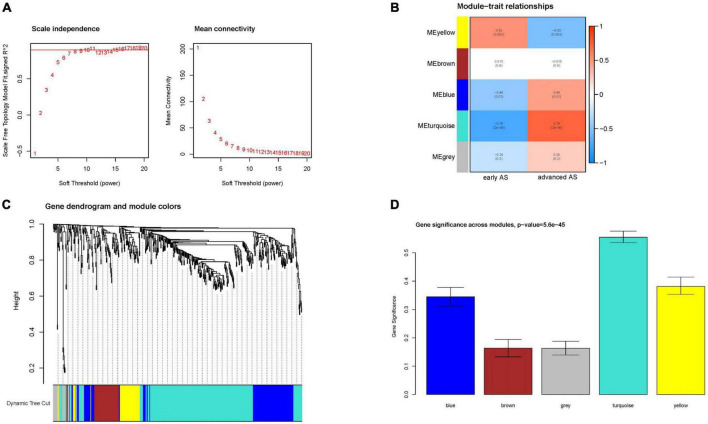
Module gene screening. **(A)** Network topology analysis. **(B)** Module-trait relationships. **(C)** Gene clustering dendrogram. **(D)** Module gene importance histogram.

To obtain the genes most associated with advanced AS, we performed a clustering analysis of early and advanced patients using the WGCNA approach. The *R*^2^ = 0.86 for the curves in the study, and a weighted network was constructed with a scale-free topological criterion (Figure 3A). We constructed a total of five co-expression modules, which were indicated by different colors (Figure 3B). We obtained turquoise modules from module-feature correlations from can get the most phase with advanced AS (MEturquoise: *r* = 0.76, *P* = 2e-16). The clustering dendrogram clustered genes with common gene expression patterns in the same color module (Figure 3C). Also, we can see that the importance of genes in the Meturquoise module is significantly higher than in the other modules (Figure 3D).

### 3.3. Enrichment analysis

**FIGURE 4:**
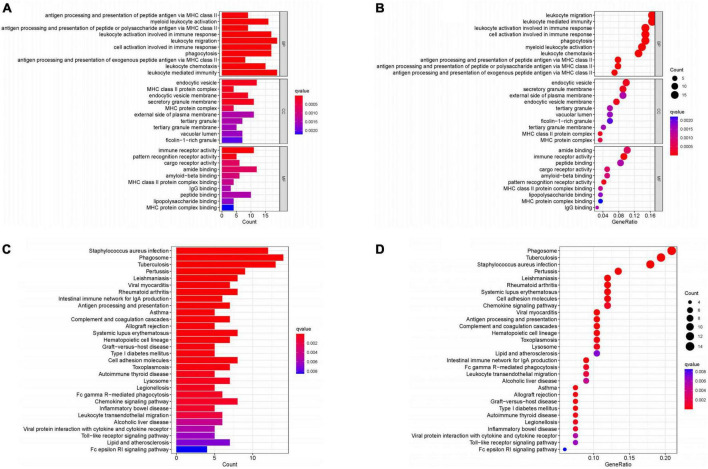
Enrichment analysis. **(A,B)** GO enrichment analysis. **(C,D)** KEGG enrichment analysis. (Histogram, the darker the color of the bar the more significant the enrichment. Bubble plot, the larger the bubble the darker the color the more significant the enrichment).

We obtained 124 core genes from the MEturquoise module based on the screening criteria MM > 0.8 and GS > 0.5 (Supplementary Table 1). The results of GO showed that these genes were mainly enriched in leukocyte mediated immunity, leukocyte migration, leukocyte activation involved in immune response, cell activation involved in immune response and phagocytosis were enriched in these functions (Figures 4A, B). While the results of KEGG can be seen Figures 4C, D, these genes were enriched in *Staphylococcus aureus* infection, Phagosome, Tuberculosis, Pertussis, and Leishmaniasis pathways.

### 3.4. Screening for key genes related to circadian regulation

**FIGURE 5:**
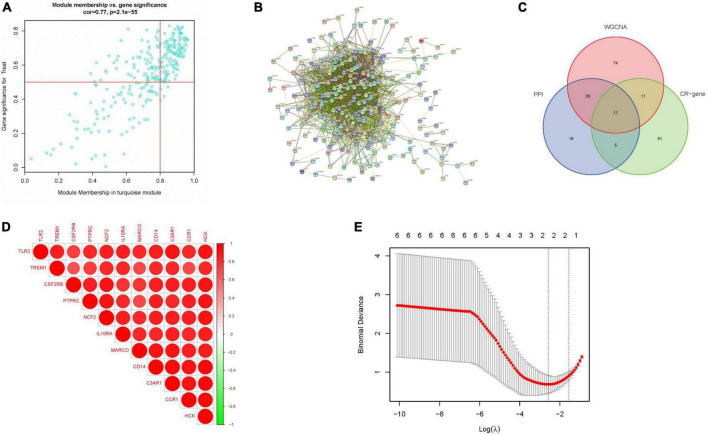
Key gene screening. **(A)** Meturquoise module gene scatter plot. **(B)** PPI of meturquoise module genes. **(C)** Veen plot of intersecting genes. **(D)** Correlation heat map of intersecting genes. **(E)** Lasso analysis. (CR-gene represents circadian regulated differential genes).

**FIGURE 6:**
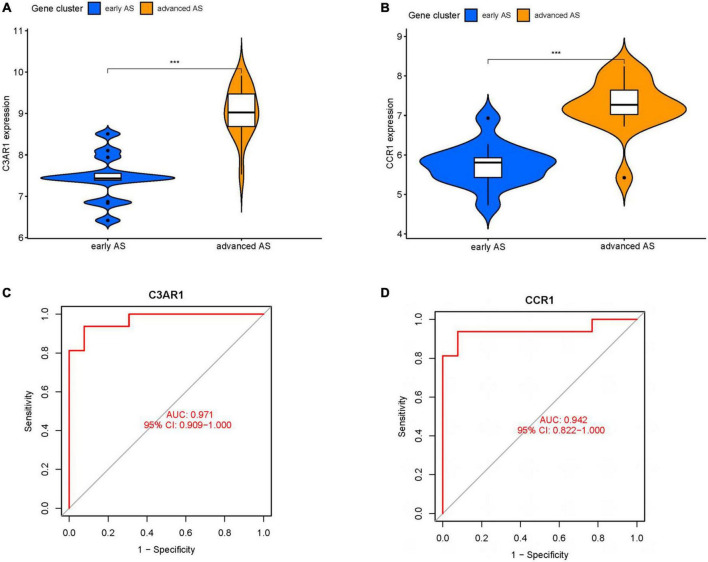
Key gene analysis. **(A)** Violin plot of expression difference of C3AR1 in early and advanced AS. **(B)** Violin plot of expression difference of CCR1 in early and advanced AS. **(C)** ROC curve of C3AR1 distinguishing early and advanced AS. **(D)** ROC curve of CCR1 distinguishing early and advanced AS. (**P < 0.01, ***P < 0.001).

We obtained 124 MEturquoise module core genes for MM > 0.8, GS > 0.5 (Figure 5A). In addition, we constructed a PPI network of 276 genes from the MEturquoise module (Figure 5B), demonstrating the reciprocal relationships among individual genes. We classified the genes with nodes > 50 as the core genes of the network, and a total of 60 core genes were obtained. To obtain the circadian regulatory genes most associated with advanced AS. We took the circadian regulatory genes of early and advanced AS differences, the core genes of MEturquoise module, and the core genes of PPI network to intersect (Figure 5C), and got a total of 11 intersecting genes. We observed the correlation of these 11 genes (Figure 5D), and it was clear that there was a significant positive correlation in the expression of these genes. Meanwhile, we further screened using lasso (Figure 5E), and finally obtained 2 key circadian regulatory genes (CCR1 and C3AR1) in advanced AS. We further went to investigate the expression differences of key genes in early and advanced AS patients, and it was clear that these two genes were significantly upregulated in advanced AS patients, which may be a response to certain *in vivo* metabolic processes (Figures 6A, B). In addition, it was clearly observed that these two key genes showed good efficacy in differentiating between early and late stage patients, with the area under the ROC curve of C3AR1 reaching 0.971 and that of CCR1 reaching 0.942 (Figures 6C, D).

### 3.5. Immunological correlation analysis

**FIGURE 7:**
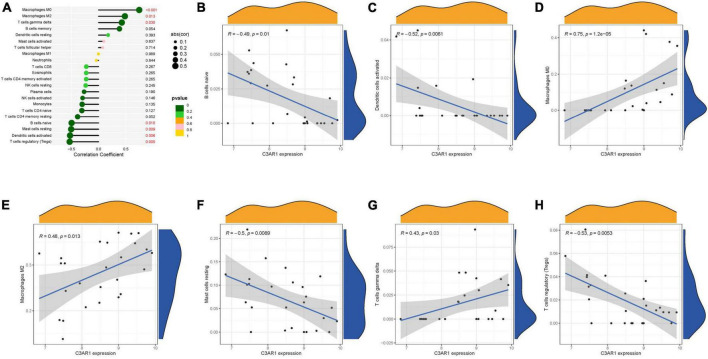
Immune correlation analysis of C3AR1. **(A)** Immune correlation lollipop chart. **(B–H)** Immune cells and C3AR1 correlation scatter plot.

**FIGURE 8:**
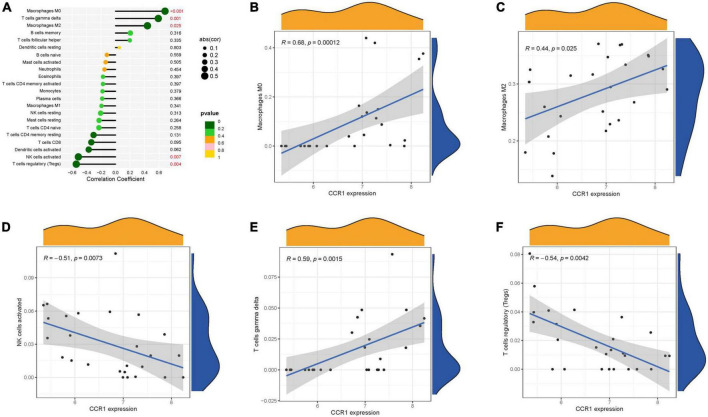
Immune correlation analysis of CCR1. **(A)** Immune correlation lollipop chart. **(B–F)** Immune cells and CCR1 correlation scatter plot.

The immune infiltration is also very correlated with AS. We investigated the correlation of key genes with immune cells. C3AR1 was positively correlated with M0 macrophages, M2 macrophages, and T cells gamma delta; and negatively correlated with B cells naïve, Mast cells resting, Dendritic cells activated, and Tregs. CCR1 was positively correlated with M0 macrophages, T cells gamma delta and M2 macrophages; and negatively correlated with NK cells activated and Tregs (Figures 7A–H) were negatively correlated (Figures 8A–F).

## 4. Discussion

To our knowledge, this is the first bioinformatics analysis that screens for key AS genes using circadian rhythms as an entry point. AS is a chronic inflammatory disease that is increasing in morbidity and mortality worldwide year by year (27). However, there are no targeted and effective treatments for AS, and its biology needs further exploration. It is estimated that approximately 50% of the risk of AS is genetically determined (28). Therefore, an in-depth exploration of AS-related genes and their involvement in pathogenesis is of great value for the targeted treatment of AS. The impact of circadian rhythms on cardiovascular disease has received increasing attention in recent years, and our analysis confirms the validity of this trend. Circadian regulation-related genes were significantly different in patients with advanced AS compared with early AS, implying that circadian regulation-related genes are closely associated with the progression of AS. We then obtained the 2 most relevant circadian regulatory genes for AS (CCR1 and C3AR1) by WGCNA and Lasso screening for analysis and validation, and found that these two genes were not only closely associated with inflammation and immune response, but their expression could also distinguish the early and late stages of AS.

Our enrichment analysis of the core genes analyzed by WGCNA revealed that these genes were mainly enriched in pathways involved in leukocyte-mediated immune responses. The activation and transport of associated leukocytes and cytokines also exhibit strong circadian rhythms and are tightly regulated by circadian rhythms. Dysregulation of circadian rhythms has been reported to directly affect leukocyte trafficking as a way to influence inflammation and immune-mediated inflammatory diseases (29), including AS. For example, disruption of circadian rhythms has been observed to significantly increase the content of damaged macrophages and increase inflammatory and oxidative stress markers in the vascular wall of AS mice (30). Circadian rhythm disruption promotes AS by modulating TLR4 to promote lipid uptake and cytokine secretion by macrophages, further activating other leukocyte-mediated inflammatory responses (31). Furthermore, it is noteworthy that leukocytes in the arterial wall have been shown to be vastly heterogeneous, with some subpopulations having a pro-inflammatory or regulatory role in the development of AS (32, 33). With the development of genome-wide association studies, mass cytometry, scRNA-seq and combined protein and RNA sequencing (genome-wide association studies, mass cytometry, scRNA-seq and combined protein, and RNA sequencing) technologies, there is considerable evidence that associated cellular and immune responses play a key role in driving chronic inflammation in the arterial vasculature in AS (34, 35). This suggests that these pathways are closely associated with the progression of AS. Our enrichment analysis and the above evidence suggest that disruption of circadian rhythms can affect AS by influencing leukocytes and their mediated immune response pathways, and that enrichment of these core genes is likely to be involved in the activation of these pathways.

Members of the G protein-coupled receptor family have been implicated in the regulation of circadian rhythms and immune responses in several studies (36). For example, they mediate the circadian effects of melatonin (37), participate in the circadian cycle and exert light resetting capacity (38). C3AR1 (G-protein-coupled receptor complement-3a receptor1), an important member of this group (39), is mainly expressed on cells such as neutrophils, monocytes and macrophages and mediates pro-inflammatory and immunomodulatory functions (40). It was found that C3AR1 expression in visceral adipose tissue was significantly higher in obese patients than in non-obese controls (41). C3AR1-knockout mice are transiently resistant to diet-induced obesity and are protected against high-fat diet-induced insulin resistance (42). This suggests that C3AR1 may be a hub between the regulation of inflammation and glucolipid metabolism. In addition, the expression of C3AR1 has been suggested to be associated with the formation of thoracic aortic aneurysms (43). A study by Propson et al. found that mice with C3AR1 knockout in vascular endothelial cells had a significantly diminished tendency toward vascular aging with age, with abnormal activation of peripheral immune cells and reduced inward flow (39). This suggests that C3AR1 is closely associated with vascular dysfunction.

The expression of CCR1 (C-C chemokine receptor type 1) has been shown to exhibit circadian oscillations (44). It is not only similar to C3AR1 in cellular origin, but both are involved in inflammatory and immune responses, regulating body metabolism and vascular responses. For example, it induces the migration of monocytes, promotes the spread of inflammatory responses and endothelial damage (45), mediates the recruitment of neutrophils to large arteries and peripheral veins, and participates in the deposition of many other inflammatory factors in the arterial endothelium (46). In addition, recent studies have shown that CCR1 is associated with hypertension, perivascular fibrosis, aneurysm formation and prognosis of cerebral hemorrhage (47–49). These are sufficient to suggest that CCR1 can be involved in a variety of pathological processes associated with vascular disease, which, in turn, are closely associated with the development of AS, especially endothelial injury, vascular inflammation.

Our immune correlation analysis showed that CCR1 and C3AR1 expression positively correlated with macrophage and T cell infiltration, and that activation of both types of cells and mediated inflammatory responses promote AS. Furthermore, AS contributed by dysfunction of these two cells has been shown to be regulated by circadian rhythms (20, 50). Infiltration of immune cells negatively associated with CCR1 and C3AR1 such as B cells, mast cells, Treg cells and NK cells on the one hand exhibit significant circadian oscillations as immune cells. On the other hand it has been shown to suppress inflammatory responses and have a protective effect on AS (51–54), so that the reduction of infiltration of these cells may, to some extent, promote the progression of AS. As for DCs, they play a dual role in the AS process due to the diversity of their isoforms and the secreted soluble factors (55). Its negative correlation with C3AR1 suggests that the pro-AS effect of C3AR1 could be achieved by inhibiting the protective role of DC in AS, and it remains to be investigated which isoforms or which specific soluble factors are at play. In conclusion, our analysis suggests that circadian rhythm disturbances are likely to affect the infiltration of these immune cells by regulating the expression of CCR1 and C3AR1 in these immune cells, which in turn affects the progression of AS. Furthermore, CCR1 and C3AR1 are not only key genes in the circadian rhythm regulatory mechanism that is closely related to immune cell infiltration, but they are also significantly more expressed in late AS samples than in early ones. They can also influence pathological changes in the vasculature by regulating the same circadian regulation-related gene VCAM1 downstream of it. For example, the C3A/C3AR signaling axis regulates VCAM1 expression, which affects peripheral immune cell infiltration and alters the functional state of the vasculature (56). In contrast, upregulation of CCR1 expression on vascular smooth muscle can promote intimal injury through VCAM1, which plays an important role in vascular dysfunction and remodeling (57).

Based on the above analysis, we hypothesize that circadian rhythm disorders can alter the expression of CCR1 and C3AR1 and participate in the development of AS via the inflammatory or immune pathways that interact with them, making CCR1 and C3AR1 promising as potential target diagnostic markers for AS. Although no studies have yet elucidated the specific mechanisms of action between these two genes and AS, our study and previous studies suggest that these two circadian-regulated genes are closely linked to the development of AS and may be novel molecular targets for the diagnosis and treatment of AS. Furthermore, since circadian rhythms can affect a variety of cardiovascular diseases, and the immune and inflammatory responses involved in these two related genes can occur in all types of cardiovascular diseases, the results of research on these two genes may be extended to a variety of cardiovascular diseases.

There are some unavoidable limitations to this study. On the one hand, all findings are based on bioinformatics techniques and lack experimental validation, further vivo and vitro experiments are needed to verify these findings. On the other hand, the sample size of this study was not very large due to the small number of available datasets related to AS in the open announcement database, which may lead to some bias in the results.

## 5. Conclusion

Through bioinformatics analysis, we obtained 2 key circadian rhythm-related genes. They have not been shown to be related to AS in previous studies, and may play an important role in circadian rhythm disorders contributing to AS. They may also be used to differentiate patients with early and advanced AS for diagnosis, becoming a new molecular target for future treatment of AS, providing a theoretical basis and a new direction for our future research. In conclusion, these 2 genes are promising to be novel molecular targets for the treatment of AS in the future. This also provides a theoretical basis and a new direction for future research.

## Data availability statement

The datasets presented in this study can be found in online repositories. The names of the repository/repositories and accession number(s) can be found in the article/Supplementary material.

## Author contributions

JY: conceptualization, methodology, formal analysis, data curation, writing—original draft, revising literature, software, and validation. HL: validation, writing—review and editing, and funding acquisition. JL: supervision. All authors contributed to the article and approved the submitted version.

## Footnotes

1
https://www.ncbi.nlm.nih.gov/geo/

2
https://cn.string-db.org/

## Supplementary material

Supplementary Table 1 MEturquoise module core genes.
